# Supplementary figures and images for: Inhibition of the amino‐acid transporter LAT1 demonstrates anti‐neoplastic activity in medulloblastoma
Source: J Cell Mol Med. 2019 Feb 19;23(4):2711–8. doi: 10.1111/jcmm.14176 (PMC6433660; doi:10.1111/jcmm.14176)

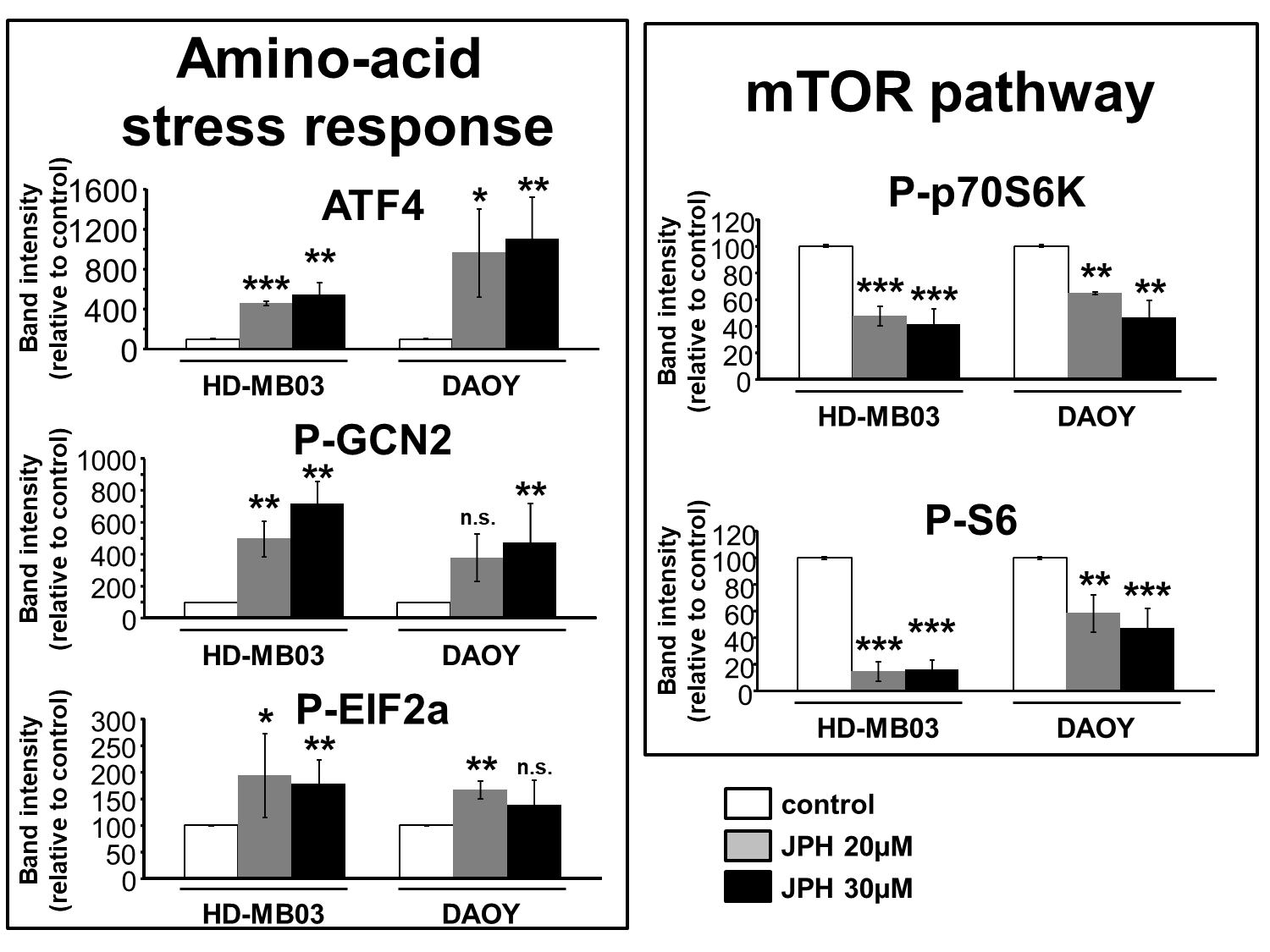

Supplement: Supplementary file 1 [file JCMM-23-2711-s001.tif]

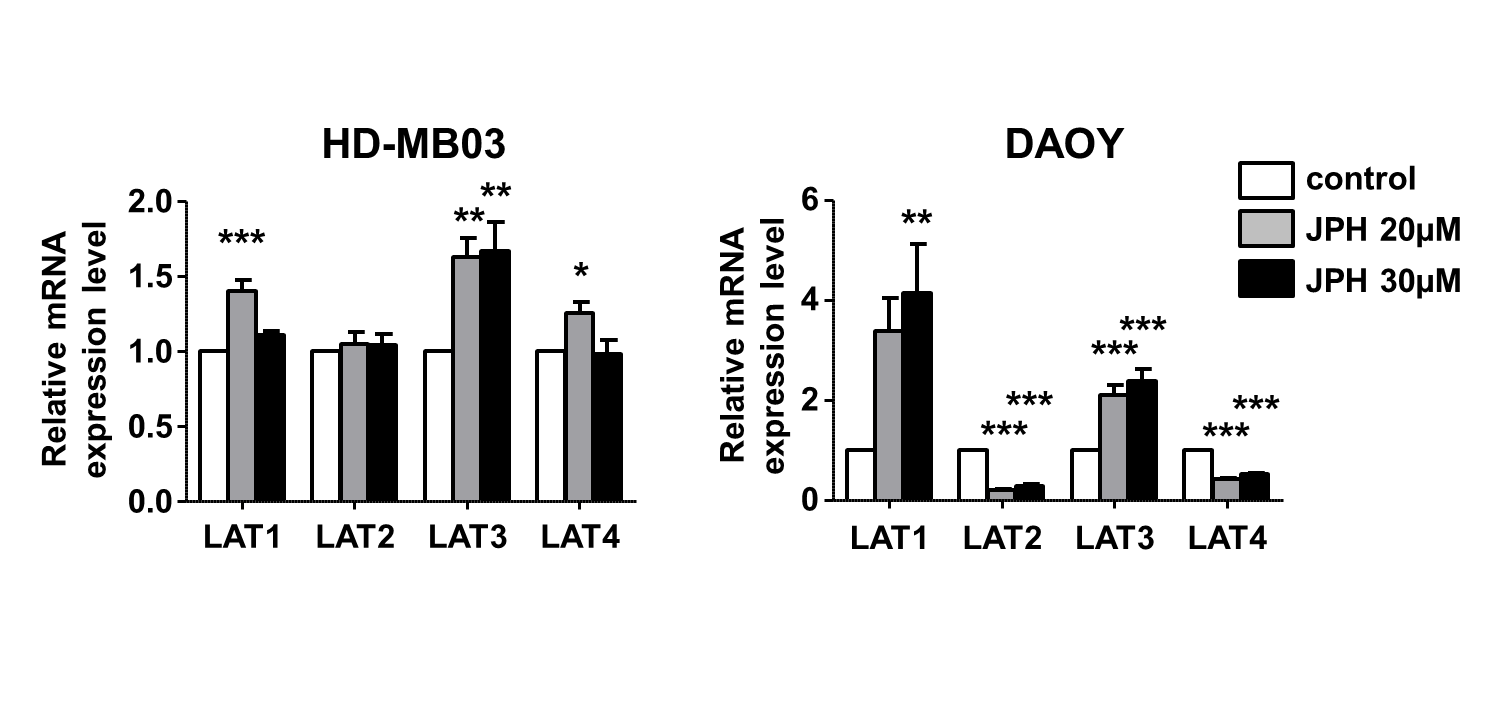

Supplement: Supplementary file 2 [file JCMM-23-2711-s002.tif]
