# Supplementary material for: Inhibition of the amino‐acid transporter LAT1 demonstrates anti‐neoplastic activity in medulloblastoma
Source: J Cell Mol Med. 2019 Feb 19;23(4):2711–8. doi: 10.1111/jcmm.14176 (PMC6433660; doi:10.1111/jcmm.14176)
Supplement: Supplementary file 3 [file JCMM-23-2711-s003.docx]

**Figure S1: Quantifications of the western-blots for AA sensing pathways.** The intensity of the bands corresponding to ATF4 (n=3), phospho-GCN2 (n=4), phospho-EIF2α (n=5), phospho-p70S6K (n=4) and phospho-S6 (n=7) were quantified and normalized to the loading control for ATF4 or to the total protein for the phosphorylated proteins. The results are expressed relative to controls (***: p<0.001, **: p<0.01, *: p<0.1; n.s.: p>0.05; Student’s t-test).

**Figure S2: Long-term JPH treatment induces modification in amino acid transporter gene expression in MB cells.** Real-time quantitative PCR analyses of LAT family gene expression levels in HD-MB03 and DAOY cells chronically exposed for 120 days to the indicated concentrations of JPH203. The results are expressed relative to control conditions and were obtained from at least three independent experiments (***: p<0.001, **: p<0.01, *: p<0.1, Student’s t-test).
